# Mr Leslie Paine MA (Oxon), OBE Formerly House Governor of the Bethlem Royal and Maudsley Hospital (1963-1985)

**DOI:** 10.1192/pb.bp.114.048058

**Published:** 2014-10

**Authors:** Gerald Russell

Leslie Paine (or Nicky as he was widely known), who has died aged 92, was, among other things, a wartime pilot who won the Russian Federation’s Ushanov Medal and Arctic Star, a distinguished rugby player, a prolific writer on health services organisation and a highly respected psychiatric hospital administrator.

He was hospital administrator and later House Governor to the Joint Bethlem Royal and Maudsley Hospital for more than 20 years. He epitomised a generation of health service managers who worked tirelessly behind the scenes to ensure doctors were left alone to exercise clinical skills and do the best for their patients in the most favourable conditions.

When he was first appointed, the Joint Hospital already enjoyed the reputation for the sound treatment of in-patients amid comfortable surroundings. Its training programme for junior psychiatrists was keenly sought after, having been established by Professor Aubrey Lewis and by the Dean, David Davies. Academic resources were, however, limited: this was a time when the Institute of Psychiatry was diminutive and accommodated wholly within the hospital buildings. They included a cramped library and a tiny lecture theatre on the Denmark Hill site.

During the same epoch the state of the Maudsley’s research achievements was similarly still quite modest. However, over the next two decades the Maudsley advanced further to become the world leader in the provision of a sophisticated educational programme. The developments were not without difficulties: there were frequent tensions between the needs of patients for continuity of care and the needs of trainees for wide experience and therefore for relatively brief attachments. Tempers flared when consultants feared patients were being short-changed. The complex system was regulated by a committee with input from both the Institute and the Joint Hospital, but the essential requirement for success was a positive attitude of good will and mutual trust. This was guaranteed through the benevolent presence of Leslie Paine who was able to succeed in negotiating between rival interests - including the often dogmatic consultant staff. Leslie developed a useful method to elicit the trust of individual clinicians. He assured them that they were the experts in their work and he was merely an administrator whose task was simply to let them get on with the job of patient care without interference.

**Figure:**
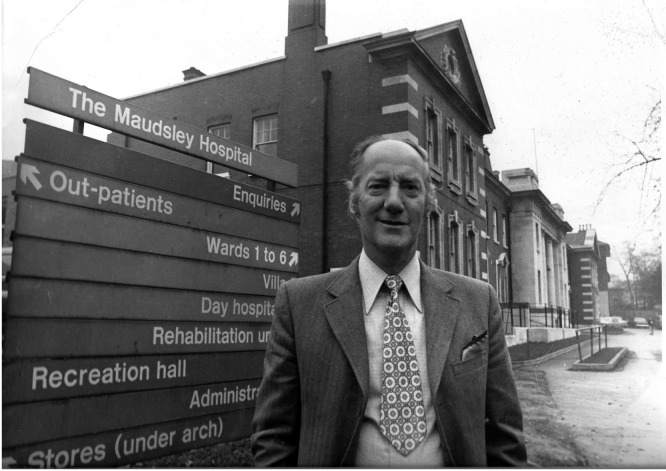
Leslie Paine at the Maudsley.

Initially it was the teaching programme that was the linchpin to the Maudsley’s enviable reputation with the research achievements appearing later and developing more gradually. In particular the Institute’s success in raising large research funds from the Medical Research Council and Wellcome Trust had to wait till the late 1980s. Prior to that point research depended on the injection of research monies from the Bethlem Royal and Maudsley Endowment Fund, a fund made possible as the result of the marriage between Bethlem and the Maudsley soon after the inception of the NHS. In the 1960s significant research grants were made from these endowment funds, supplemented with monies from the Department of Health. Again there was a need for a careful balance between the legitimate clinical claims for such money and the need to support research. At this time, Leslie Paine’s role as House Governor was crucial in managing tensions between the various spheres of activity.

Subsequently the contribution to research from the endowment funds was reduced in order to provide money for much needed clinical developments. This diminution of research monies could have had disastrous effects on the early research endeavours of young clinicians, had it not been for mitigating factors. One important such factor was the establishment of the Psychiatry Research Trust in 1980. Leslie Paine was its Director from 1986 until the end of his life, promoting fund-raising to provide grants for young researchers working in the field of mental health and brain disease.

Leslie Paine was born in Bath in 1921 and educated at the City of Bath School. During the Second World War he served in the Royal Navy as a pilot in the Fleet Air Arm. He described his wartime experiences in a gripping book *Memoirs of a Swordfish Man,* published shortly before he died. The title refers to the Fairey Swordfish, a torpedo-bomber biplane that Leslie described as one of the most successful wartime aircraft. In his book he provides a modest account of how he took part in a night strike against enemy shipping in the Norwegian fjords, in January 1945. When the action was over he and his observer found that they had lost contact with the beacon ship, the Nairana. They were close to despair when the observer spotted another aircraft. He flashed with his old Aldis lamp a request to be guided home, which had the desired effect. In February 1945 Leslie sailed with the Nairana and its complement of Swordfish escorting a large convoy of merchantmen conveying war supplies to Russia. In spite of atrocious weather and enemy action, most of the convoy ships reached Russia and returned to Britain safely.

After the war Leslie read English at Pembroke College, Oxford. He later enrolled in the King’s Fund 2-year course to train hospital administrators. He was a keen and talented sportsman who won a half-blue for rowing at Oxford and played rugby for Bath and Rosslyn Park. Later he was a gifted, stylish tennis player. He wrote prolifically and his book *Hospitals and the Healthcare Revolution* (1988) was translated into several languages. He frequently presented papers at conferences such as those run by the King’s Fund, the World Health Organization and the Institute of Psychiatry. He also wrote regular articles on rugby and tennis for the Times and Cambridge Daily News. He was appointed OBE in 1970.

Leslie had a strong sense of compassion and a kindly sense of humour. He was known by his family, many friends and colleagues as a gentle gentleman. Leslie was previously married to Thelma (d. 1978) and Sally (d. 2006). In recent years Christine Lutman was his partner and provided many details of this obituary. She was with him when he died peacefully aged 92. He is also survived by his daughter, Bryony, and her family.

Leslie Paine, born 4 October 1921, died 2 December 2013.

